# Service-Based RAN User Plane Decoupling and Orchestration via ComBERT for AI AgentServices

**DOI:** 10.3390/s26175318

**Published:** 2026-08-22

**Authors:** Haiyu Ding, Shangyuan Du, Xin Sun, Xiangyu Guo, Chunjing Yuan, Lin Tian, Shuyuan Zhang, Jing Jin

**Affiliations:** 1 China Mobile Research Institute, Beijing 100053, China; 2 Institute of Computing Technology, Chinese Academy of Sciences, Beijing 100190, China; 3 China Mobile Group Henan Co., Ltd., Zhengzhou Branch, Zhengzhou 450008, China; 4 Nanjing Institute of InforSuperBahn, Nanjing 210008, China

**Keywords:** 6G, service-based RAN, user plane decoupling, ComBERT, service orchestration

## Abstract

The rapid development of large model-driven agent applications, such as digital assistants and robots, requires 6G radio access networks (RAN) to deliver enhanced flexibility, adaptability, and low-latency capabilities. However, the existing RAN user plane (UP) architecture suffers from coarse decoupling granularity and significant cross-layer functional redundancy. These limitations severely hinder the on-demand orchestration and dynamic reconfiguration required by heterogeneous agent services. To address these challenges, this paper proposes a ComBERT-driven service-based RAN UP decoupling method, specifically targeting the functional coupling and redundancy between the PDCP and RLC sublayers. First, we develop a domain-specific language model, ComBERT, by pre-training a BERT model on a 3GPP protocol corpus and fine-tuning it on text-matching tasks to deeply comprehend protocol semantics. Subsequently, ComBERT is utilized to extract semantic features from UP functional components, employing a sliding window mechanism to overcome truncation in lengthy protocol texts and using cosine similarity to measure functional relevance. Finally, a threshold-based fusion algorithm is designed to identify and merge cross-layer redundant functions, thereby forming independent service units with distinct responsibilities. These fused units serve as the basic building blocks for scenario-specific orchestration. Simulation results demonstrate that the proposed method reduces the number of UP components by 12.5%, 18.7%, and 18.2% in eMBB, URLLC, and mMTC scenarios, respectively. Simultaneously, it decreases average processing delays by 7.9%, 10.2%, and 11.0% across these respective scenarios. Ultimately, this approach effectively improves the lightweight deployment, processing efficiency, and reconfiguration capabilities of the service-based UP, providing a crucial foundation for on-demand service orchestration in 6G networks tailored to agent services.

## 1. Introduction

Driven by the rapid development of large model technology, the service targets of networks are gradually expanding from traditional mobile Internet services to novel agent applications, including digital assistants, embodied robots, and unmanned systems [1]. Compared to traditional text, voice, and video services, agent services exhibit distinct characteristics in terms of service organization, interaction processes, and resource requirements [2]. These agent services inherently feature continuous interaction, task-driven operations, and dynamic closed loops, making them highly sensitive to network latency, processing efficiency, and service continuity [3]. Furthermore, the execution of agent services frequently involves the uploading of sensory information, the downloading of inference results, collaborative edge processing, and multi-stage task feedback. Consequently, the network must deliver not only basic connectivity but also enhanced capabilities in service organization, functional adaptation, and resource coordination. Against this backdrop, 6G networks are evolving from traditional communication networks into information service networks that integrate connectivity, computing, data, and intelligent capabilities [4]. As the crucial segment closest to end-users and service endpoints, the architectural design of the RAN directly determines the carrying efficiency and service quality of agent applications [5,6].

In a 6G RAN tailored to agent services, the UP should no longer serve merely as a rigid data transmission pipeline within a layered protocol stack [7]. Instead, it must possess the capabilities for flexible composition, dynamic tailoring, and lightweight deployment based on specific task requirements. The recent advancement of the service-based architecture (SBA) in the core network offers vital insights for the evolution of the RAN. By decomposing network functions into independently invocable service units with distinct responsibilities and enabling flexible composition and on-demand deployment via standardized interfaces, the SBA has demonstrated remarkable openness and scalability within the core network [8]. Inspired by this paradigm, introducing the service-based design philosophy into the UP of the RAN can significantly enhance the network adaptability to heterogeneous agent services. This integration establishes a foundational framework for the subsequent coordination of connectivity and computing, task-driven service orchestration, and the continuous evolution of service quality assurance capabilities [9].

AI-agent services serve as the primary architectural motivation for this work rather than as an experimentally evaluated application scenario. Accordingly, the present evaluation is limited to protocol-level component reduction and simulated processing delay under the representative eMBB, URLLC, and mMTC traffic profiles; it does not evaluate sensing-data generation, edge-inference latency, control-loop timing, or task-level performance.

According to 3GPP protocol specifications, the UP of the RAN consists of multiple functional sublayers including SDAP, PDCP, RLC, MAC, and PHY. The SDAP sublayer performs relatively simple functions, while the MAC and PHY sublayers possess stringent real-time requirements and clear data processing boundaries, making them relatively easy to implement as independent services [10]. Conversely, the PDCP and RLC sublayers exhibit strong intra-layer coupling and inter-layer dependencies, and certain internal functions display obvious cross-layer isomorphism and redundancy. Mechanisms including sequence number maintenance, duplicate detection, and status feedback are implemented separately within both sublayers [11]. Although this functional arrangement suits traditional layered protocol architectures, it introduces additional processing overhead and resource redundancy during service-based reconfiguration. Furthermore, it hinders the flexible tailoring and rapid orchestration required to meet varying service demands. In the context of 6G access networks tailored to agent applications, such a coarse-grained organization of the UP severely limits network capabilities in achieving low-latency processing, efficient resource utilization, and on-demand functional reconstruction [12]. Therefore, how to break through traditional hierarchical boundaries to achieve more fine-grained decoupling and reorganization of internal user plane functions constitutes a key challenge in the evolution of service-based RAN.

Numerous researchers have proposed various service-based approaches. Wang et al. introduced the CUPS-E2E network slicing scheme, which divides base stations into Control Base Stations (CBS) and Traffic Base Stations (TBS) to achieve collaborative support for wide coverage of the Control Plane (CP) and diverse service requirements of the UP [13]. Du et al. proposed a Converged Service-Based Architecture (cSBA), presenting optional converged service definitions, service-based interface implementations, and a converged service framework to enable efficient decoupling of the access, control, and data planes [14]. Yuan et al. put forward a service-based RAN architecture capable of deploying decoupled RAN functions and customizing them according to specific tasks [15]. Schmidt et al. introduced a containerized service-based RAN architecture that decouples the CP and UP of Distributed Units (DUs) to facilitate function customization and expansion [16]. Li et al. designed a 6G service-based RAN architecture based on the principle of “high cohesion, low coupling,” proposing a service-based data interaction model for the UP to transform the architecture from a layered structure to a service-based chain and support dynamic module invocation [17]. Zong et al. proposed a service-based RAN architecture and redefined corresponding functions. Aiming at the insufficient flexibility and openness of the current RAN architecture (which evolves functions in units of CU-CP, CU-UP, and DU) [18]. Song et al. proposed a microservice approach to the vDU user plane, evaluating the microservice design of RAN software components based on cloud-native principles and cloudified RAN [19]. The existing research has provided an essential foundation for the service enablement and UP reconstruction of radio access networks. However, most of these studies remain confined to splitting the UP at the holistic or sublayer level. They pay insufficient attention to the semantic relationships among fine-grained functions within protocols, cross-layer functional redundancy, and lightweight reorganization tailored to intelligent services. Consequently, although current service-based designs have improved modularity, they still fail to fully resolve the limitations imposed by rigid internal functional boundaries and cross-layer redundancy on flexible orchestration capabilities.

### 1.1. Domain-Adaptive NLP and Telecom Language Models

The semantic analysis of protocol specifications relies heavily on Natural Language Processing (NLP) techniques. While general-purpose language models such as BERT and Sentence-BERT (SBERT) excel at generic text similarity tasks, they often struggle with specialized technical-domain terminology and sublayer-specific operational semantics. Recently, domain-adaptive pre-training, as represented by SciBERT and related approaches, has demonstrated significant advantages in understanding specialized corpora [20,21]. SBERT produces semantically meaningful sentence embeddings suitable for cosine-similarity retrieval [22]. In the telecommunications domain, adapting pre-trained transformers to 3GPP documents enables fine-grained modeling of functional dependencies that traditional lexical matching, such as TF-IDF or keyword matching, fails to capture. These studies provide the methodological basis for ComBERT. Accordingly, the contribution of this work lies not in proposing a new Transformer architecture but in applying domain-adapted semantic analysis to protocol-component candidate generation and constraint-aware PDCP/RLC reconstruction.

### 1.2. Comparison with Traditional RAN Functional Splits

Traditional functional-splitting approaches in RAN, including CU/DU and CU-CP/CU-UP decomposition, O-RAN disaggregation, and cloud-native microservice architectures, focus primarily on macro-level partitioning along fixed protocol or deployment boundaries. The O-RAN service-based RAN study recommends retaining backward-compatible CU/DU and CP/UP baselines while investigating finer service granularity and accounting for the latency cost of user-plane service interfaces [23]. Although these approaches facilitate modular software deployment, they do not directly identify fine-grained semantic and operational redundancy between PDCP and RLC procedures. In contrast, the proposed component model operates within a selected UP deployment domain and examines protocol functions below the conventional sublayer granularity. It complements rather than replaces existing F1, E1, and open-fronthaul boundaries, and permits cross-layer consolidation only when protocol execution constraints remain satisfied.

Building upon these considerations and addressing the demands of 6G networks for the flexible accommodation and efficient processing of agent services, this paper investigates fine-grained UP decoupling in service-based RAN. The research primarily focuses on the identification, correlation analysis, and fusion reconstruction of intra-layer and cross-layer functions between the PDCP and RLC sublayers. A ComBERT-driven service-based UP decoupling scheme is thereby proposed. The main innovations of this paper are summarized as follows:

1. A fine-grained UP component modeling method based on protocol semantics is proposed. By defining and textually representing the functions of the PDCP and RLC sublayers as components, this paper further liberates UP functions from traditional rigid hierarchical structures. This liberation establishes a foundation for subsequent flexible composition, dynamic tailoring, and service-based orchestration according to specific service requirements.
2. A domain-specific language model named ComBERT is constructed for understanding the semantics of communication protocols. By conducting domain-specific pre-training on 3GPP protocol texts and fine-tuning the model with text-matching tasks, the capability of the model to understand professional communication terminology, functional descriptions, and contextual relationships is significantly enhanced. Compared with directly employing general-purpose language models, this method is more suitable for characterizing the internal functional relationships of communication protocols. It provides robust support for the functional identification and semantic analysis of protocol components, laying a foundation for future integration into task-driven agent service pipelines.
3. A cross-layer functional fusion and reconstruction method for the UP based on semantic relevance is proposed. By constructing a component semantic representation space and quantifying the correlation degree among different functions, this paper achieves the identification and fusion of cross-layer redundant functions between the PDCP and RLC sublayers. The resulting fused service units serve as the foundation for scenario-specific service orchestration, where they are composed into processing chains for eMBB, URLLC, and mMTC applications. This approach breaks through the restrictions imposed by traditional layered protocol boundaries on functional organization methods. The reconstructed UP service units possess clearer responsibility boundaries and higher organizational flexibility. These improvements are evaluated at the protocol level under eMBB, URLLC, and mMTC traffic profiles; their effect on end-to-end AI-agent task performance is outside the scope of the present study.

The subsequent chapters are organized as follows: Section 2 introduces the service-based UP architecture; Section 3 elaborates on UP modeling, including the definition of components and processes; Section 4 details the construction of the UP component language model (ComBERT); Section 5 presents the UP component fusion and reconstruction strategy; Section 6 evaluates the performance of the proposed scheme through simulation experiments.

## 2. User Plane Service-Based Architecture

To meet the demands of diverse vertical application scenarios, the service-based UP must decouple tightly integrated functional relationships and abandon the traditional monolithic protocol architecture, thereby enabling the construction of a service-based system that can be deployed independently. This transformation introduces notable advantages in terms of agility and adaptability, enhancing the dynamic capabilities of 6G networks.

**Figure 1:**
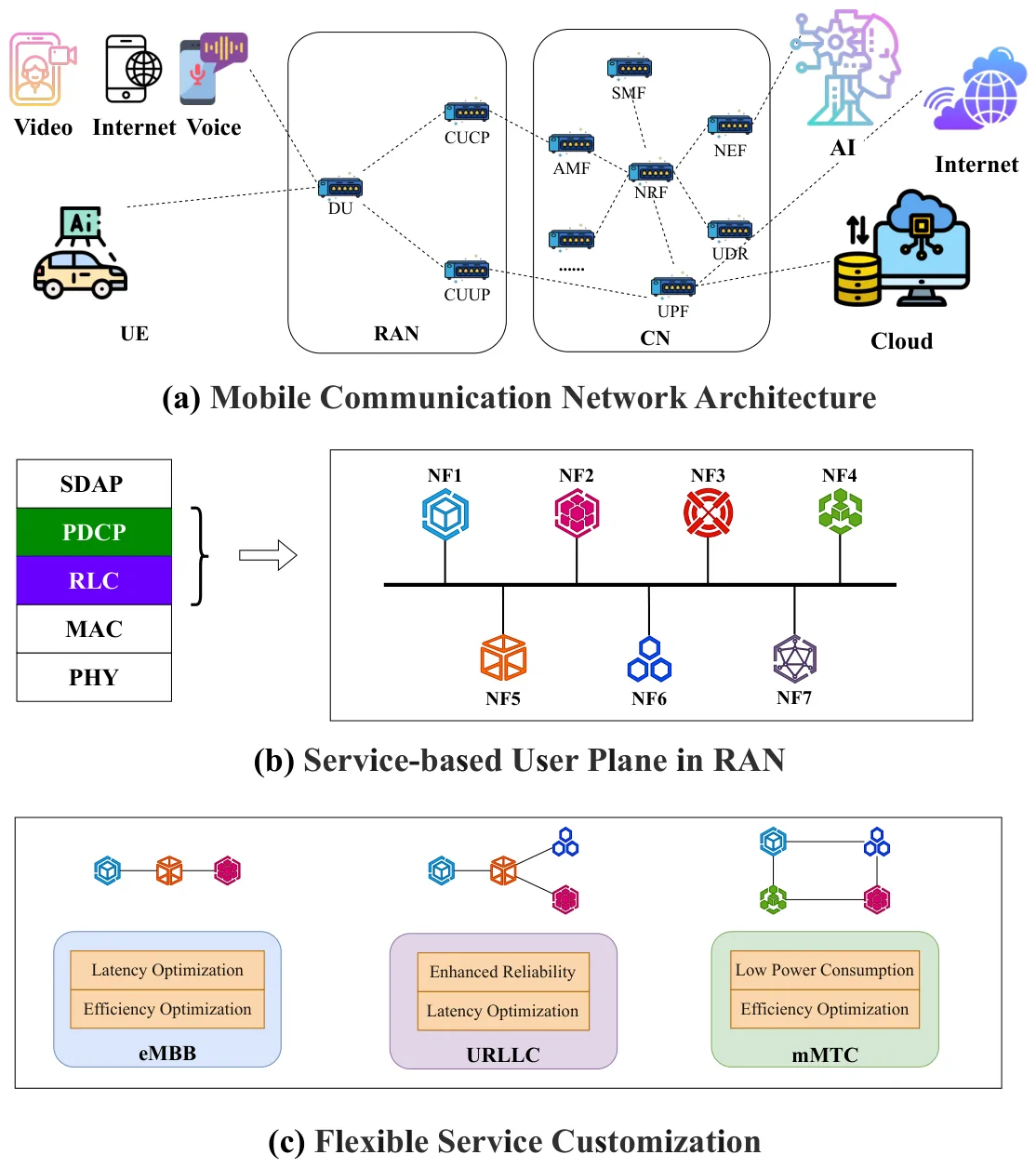
Service-based UP architecture.

As illustrated in Figure 1, the service-based UP architecture decouples protocol layers into independent functional services, enabling flexible composition and on-demand orchestration. The UP decouples the functions of each layer into a set of independent services, with each being designed to perform a single, well-defined responsibility. The UP is decoupled into layers, including SDAP, PDCP, RLC, MAC, and PHY. With consideration of the coupling relationship between PDCP and RLC, a more fine-grained decoupling is implemented to enable the reconstruction and lightweight design of both the PDCP and RLC layers. The decoupled UP enables flexible combination and orchestration of services tailored to the specific requirements and objectives of diverse vertical industries, significantly improving the network’s flexibility and customizability.

## 3. UP Modeling

### 3.1. Component

In this work, the minimum processing unit of the UP is defined as a component based on context. Context refers to the relevant information and environment required when a functional instance executes a request or operation, including data and states related to the request or operation. The context determines a component, the operation performed on the context, and the Status of the operation. Components for the PDCP and RLC sublayers of the UP were extracted from 3GPP TS 38.323 (PDCP) [24] and TS 38.322 (RLC) [25] protocol documents. Textual descriptions of all components were obtained to build a UP component set:

(1) $$A=\left\{A_{i}|i=1,2,\ldots ,n\right\},\;A_{i}=\langle {\mathrm{Name}}_{i},{\mathrm{Description}}_{i}\rangle$$

where $A_{i}$ represents a component, consisting of the component name ${\mathrm{Name}}_{i}$ and the textual description ${\mathrm{Description}}_{i}$ of the component in the protocol. All components of PDCP and RLC constitute the UP component set *A*. As shown in Table 1, this work only considers 13 PDCP components and 9 RLC components. Component extraction targets explicitly defined 3GPP functions with independent logic and standalone implementability, specifically excluding those that are tightly coupled or lack clear boundaries.

### 3.2. Process

A process represents the sequential packet processing path in the UP. Consequently, a specific process $P_{\mathrm{type}}$ is modeled as a chain of components:

(2) $$P_{\mathrm{type}}=\left\{A_{\mathrm{type},1},A_{\mathrm{type},2},\ldots ,A_{\mathrm{type},n_{P}}\right\}$$

where type denotes the specific scenario category, such as typical service scenarios like eMBB, URLLC, and mMTC. $n_{P}$ is the number of components in the process, and the sequence $P_{\mathrm{type}}$ indicates the components and their processing order for each scenario.

Once the business scenario is determined, the components in the process $P_{\mathrm{type}}$ are fixed. The processing time $T_{\mathrm{type}}$ of process $P_{\mathrm{type}}$ can be defined as the sum of the processing times of all components in the chain:

(3) $$T_{\mathrm{type}}=\sum_{i=1}^{n_{P}}t_{\mathrm{type},i}$$

where $t_{\mathrm{type},i}$ is the processing time of component $A_{\mathrm{type},i}$.

Service orchestration composes independent service units into scenario-specific processing chains according to task requirements. For each scenario (eMBB, URLLC, or mMTC), the fused service units generated from Section 5 are composed into a specific processing chain $P_{\mathrm{type}}$. The orchestration granularity is determined by the fusion results: finer fusion produces more lightweight units, while coarser fusion reduces orchestration complexity.

## 4. Construction of ComBERT

ComBERT is evaluated on protocol-specific similarity tasks and semantic-driven UP reconstruction. BERT encodes component descriptions $A_{i}$ into text vectors $v_{i}$:

(4) $$v_{i}=\mathrm{BERT}\left({\mathrm{Description}}_{i}\right)\in R^{768}$$

Input the textual description of component $A_{i}$, ${\mathrm{Description}}_{i}$, into BERT to obtain the text vector $v_{i}$ of the component, which is a 768-dimensional vector.

**Figure 2:**
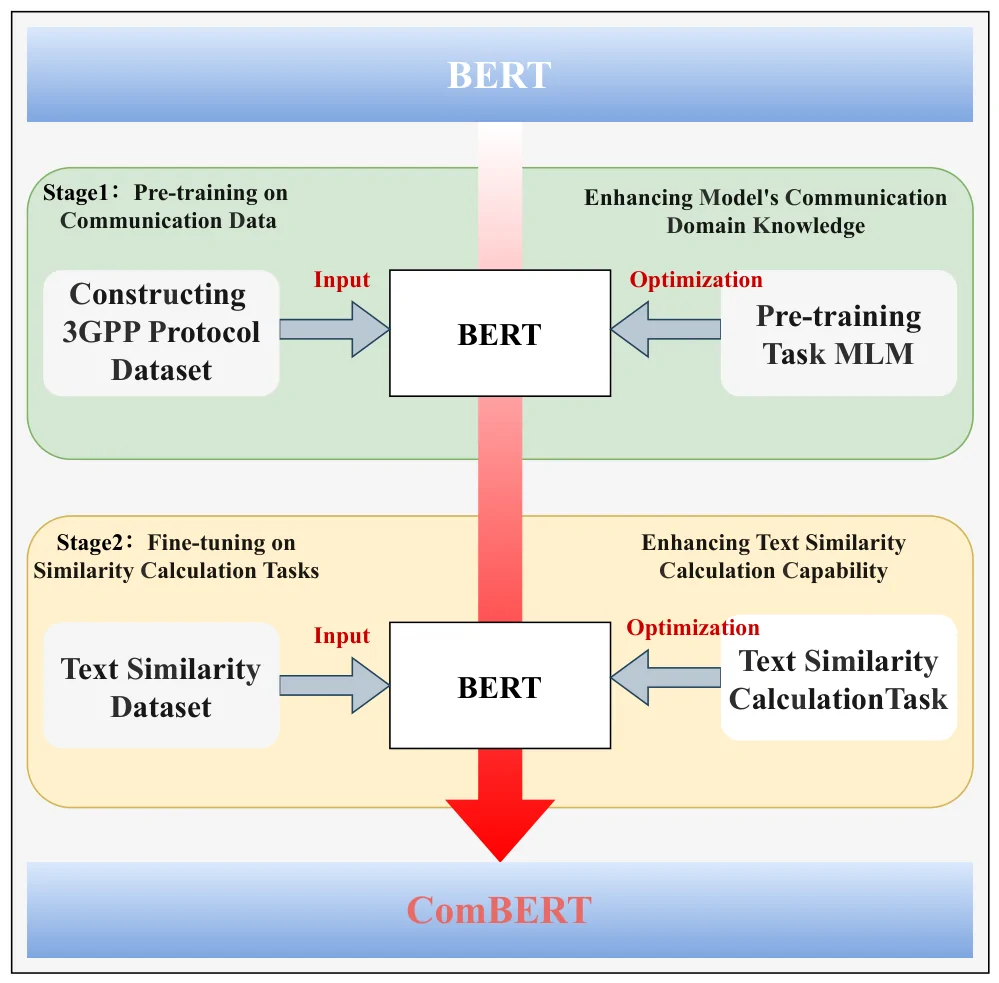
BERT improvements.

The original BERT is a general text pre-trained model, but its original form has certain limitations in text-matching degree calculation tasks and knowledge within the communication domain. To enhance the performance of BERT in communication domain knowledge and text-matching degree tasks, this work addresses two key aspects, as illustrated in Figure 2. Figure 2 illustrates the two-stage enhancement of BERT, including domain-adaptive pre-training and similarity task fine-tuning.

### 4.1. Fine-Tuning BERT on Communication Domain Knowledge

To address the limitation that the original pre-trained model BERT lacks telecom-specific knowledge, this section presents a solution. By constructing a 3GPP protocol text dataset, standard protocol documents are converted into a format compatible with BERT’s input requirements. Subsequently, BERT is pre-trained using the Masked Language Modeling (MLM) task, thereby enhancing its understanding of telecom-specific text and enriching its knowledge in the field of telecom protocols.

#### 4.1.1. Construction of the Telecommunication Domain Dataset

The 3GPP protocol standards, which are composed of Word documents, cannot be used directly as input for BERT. Therefore, the Word document data from 3GPP undergoes cleaning, to transform the 3GPP documents into pure text data processable by BERT. Firstly, the docx files are converted into plain text format, and the text content is extracted. The original 3GPP standard documents are formatted and typeset in a specific manner. However, these elements are not required by BERT; thus, the table of contents, figures, labels, and chapter titles are removed from the original documents. Additionally, surplus blank lines and line breaks are deleted. Next, the text paragraphs are split into sentences, and paragraphs exceeding a certain length are truncated. Ultimately, the 3GPP standard documents are converted into a telecommunication pre-training dataset compatible with BERT.The constructed dataset is based on 3GPP TS 38.323 [24] and TS 38.322 [25] (Release 16), including approximately 50,000 sentences and 1.2 million tokens. The dataset is split into training, validation, and test sets with a ratio of 8:1:1. A domain-specific tokenizer with a vocabulary size of 32,000 is adopted.

#### 4.1.2. BERT Domain-Adaptive Pre-Training

Domain-adaptive pretraining is performed using the MLM objective to enhance telecom-specific semantic understanding. A masking strategy with a 15% probability is applied, including replacement, random substitution, and unchanged tokens.

In the domain-adaptive fine-tuning phase, this work continues to pre-train BERT on telecommunication-specific data using the MLM task. The implementation includes the following key steps: building a tokenizer based on a telecommunication-specific vocabulary, protecting subwords for technical terms such as “PDCP” and “RLC”, and adding them to the vocabulary. Mask technical terms (e.g., protocol names and functions) with a 10% probability, while maintaining an overall masking rate of 15% to inject domain knowledge efficiently. For example, the original sentence [“The PDCP layer is responsible for data header compression.”] becomes [“The [MASK] layer is responsible for data header compression.”] after masking. With the setting of a total masking rate of 15%, the masking probability is allocated through the following equation:

(5) $$P_{\mathrm{mask},\mathrm{type}}=\left\{\begin{array}{cc} 0.8, & \left[\mathrm{MASK}\right]\;\mathrm{replacement},\\ 0.1, & \mathrm{random}\;\mathrm{replacement},\\ 0.1, & \mathrm{unchanged}. \end{array}\right.$$

This mechanism uses 10% random replacement. This boosts the model’s generalization, allowing it to reason accurately even when encountering erroneous information, rather than relying excessively on the [mask] token. It also enhances the model’s robustness to noise. Additionally, by keeping 10% of the words unchanged, the approach reduces the mismatch between training and inference. The model can thus reasonably model contexts even when it sees correct words.

The cross-entropy loss at masked positions is calculated using the actual inputs and predicted outputs:

(6) $$L_{\mathrm{MLM}}=-\sum_{i\in M}\log P\left(w_{i}|h_{i}\right)$$

where *M* denotes the set of masked positions, $h_{i}$ represents the output vector of BERT at the masked position, $w_{i}$ is the ground-truth word at the masked position, and the conditional probability $P(w_{i}|h_{i})$ denotes the probability of predicting the ground-truth vocabulary $w_{i}$ based on BERT’s output. By summing the negative log-likelihoods of all masked positions in *M*, the model is forced to learn the ability to restore domain terms from interferences ([MASK]/random words). After calculating the loss, the gradient of each parameter in the model can be obtained to achieve model update and tuning. Through continuous training and iteration on the communication dataset, the loss is minimized as much as possible. The cross-entropy loss $L_{\mathrm{MLM}}$ represents the model’s understanding of the communication dataset—the smaller the loss value, the deeper the model’s knowledge of various terms in the communication dataset.

When pre-training BERT using the MLM task with the communication dataset, the loss generated during training, denoted as $L_{\mathrm{MLM}}$, represents the discrepancy between the predicted results and the ground truth. A smaller $L_{\mathrm{MLM}}$ indicates that BERT has a more precise understanding of the communication pre-training dataset. After sufficient training, $L_{\mathrm{MLM}}$ on the communication pre-training dataset will gradually approach zero, signifying that the BERT model can accurately predict the masked words in 3GPP protocol texts (similar to a cloze test). This indicates that the model has adapted to the communication-domain text distribution and learned protocol-related contextual patterns.

### 4.2. Training of BERT for Text-Matching Degree Tasks

Even after being enhanced with knowledge from the telecommunication domain, BERT remains a language model and cannot inherently compute text-matching degree between two texts. To enable this capability, this work enhances the BERT model by defining a new loss function (optimization objective) and conducting optimization and training on a text-matching degree dataset. This process enables the model to develop the capability to compute text-matching degree.

#### 4.2.1. Dataset Preparation

To enable the existing BERT model to compute text-matching degree, it needs to be trained on a text-matching degree dataset. Each entry in this dataset consists of a text pair and a similarity score, formatted as:

(7) $$\left\{{\mathrm{text}}_{1},{\mathrm{text}}_{2},\mathrm{score}\right\},\;\mathrm{score}\in \left[0,1\right]$$

Here, ${\mathrm{text}}_{1}$ and ${\mathrm{text}}_{2}$ form a text pair, and score indicates their similarity, with higher values meaning greater similarity. For fine-tuning BERT, this work selects the general-purpose STS-B dataset, which is dedicated to text-matching degree. To better align with the communication domain, a small set of protocol-based text pairs is additionally constructed for further fine-tuning, at which point similarity scores are assigned according to functional relevance in 3GPP specifications.

To tailor ComBERT for protocol semantic matching, a specialized telecommunication text-pair dataset was constructed from the 3GPP TS 38.323 [24] and TS 38.322 [25] specifications. The dataset comprises 1200 fine-grained protocol-description pairs. To ensure annotation objectivity and dataset quality, each text pair was independently evaluated by three domain experts in 6G wireless communication architectures and assigned a semantic similarity score score∈[0,1] according to functional equivalence and data-flow interdependencies. Inter-annotator consistency was assessed through the average pairwise Pearson correlation coefficient, yielding ρ=0.87. The annotated pairs supplement STS-B with relations involving identical responsibility, partial functional overlap, dependency without redundancy, and operational incompatibility. To reduce information leakage, pairs derived from the same source clause were assigned to the same data split.

#### 4.2.2. Text Encoding

BERT has a maximum input length of 512. Directly inputting the lengthy text description of UP components can cause truncation, as only the first 512 tokens are used for text vector calculation, resulting in inaccurate results. To address this, this work employs the sliding-window method for processing long texts.

To handle long texts exceeding BERT’s input limit, a sliding-window strategy is adopted to preserve contextual information and aggregate representations across segments.

#### 4.2.3. Downstream Task Construction for Text-Matching Degree

A regression-based downstream task is designed to compute similarity scores using the [CLS] representations of two inputs.

After obtaining the text vectors, a fully connected layer, Linear, is added after BERT as the downstream task for calculating text-matching degree. The calculation process of the similarity between two texts is as follows:

Where the text Description of the two components are independently input into the BERT model. The [CLS] vector in BERT’s encoder output contains the complete contextual information and semantic features of the text, which is a 768-dimensional vector serving as the input to the fully connected layer in the downstream task. In the downstream task, the two [CLS] vectors are first mapped by a fully connected layer into a 4096-dimensional vector, then transformed by an output layer into a 1-dimensional vector, and finally processed by the sigmoid function to convert the output into a value between [0, 1], the similarity score of the text descriptions of the two components.

(8) $$\left\{\begin{array}{c} {\mathrm{CLS}}_{1}=\mathrm{BERT}\left({\mathrm{Description}}_{1}\right),\\ {\mathrm{CLS}}_{2}=\mathrm{BERT}\left({\mathrm{Description}}_{2}\right),\\ \mathrm{score}=\mathrm{sigmoid}\;\left(\mathrm{Linear}({\mathrm{CLS}}_{1}+{\mathrm{CLS}}_{2})\right). \end{array}\right.$$

## 5. Fusion and Reconstruction of UP Components

This section presents the component fusion operation, which identifies and merges cross-layer redundant functions between PDCP and RLC sublayers into independent service units.

We developed ComBERT by pre-training BERT on 3GPP documents and fine-tuning it on a similarity dataset, enabling accurate text-matching within the communication domain. However, semantic similarity only identifies candidate components for fusion, rather than guaranteeing functional mergeability or protocol correctness. To ensure that the reconstructed UP remains implementable, logically consistent, and designed to reduce the risk of non-compliant fusion, the final fusion decisions are governed by strict practical constraints: (1) latency-critical functions must not incur additional delay; (2) stateful functions require logically independent or unifiable state machines; and (3) security functions (e.g., encryption) strictly prohibit cross-layer merging.

Operationally, merging means implementing two compatible protocol procedures in one deployable service unit while preserving their externally observable inputs, outputs, state transitions, ordering, and failure behavior. It does not mean deleting either standards-mandated behavior. To screen out candidates that violate the encoded constraints, a pair of candidate components, $A_{i}$ and $A_{j}$, is eligible for cross-layer fusion if and only if it satisfies the following multi-attribute Boolean predicate:

(9) $$\begin{array}{cc} \mathrm{Fuse}(A_{i},A_{j})= & \left(M(A_{i},A_{j})>\theta \right)\wedge \neg \mathrm{SecCrit}\left(A_{i},A_{j}\right)\\ \; & \wedge \mathrm{StateCompat}\left(A_{i},A_{j}\right)\wedge \neg \mathrm{StrictDelay}\left(A_{i},A_{j}\right), \end{array}$$

where $\mathrm{SecCrit}(A_{i},A_{j})$ is evaluated as true if either component involves a security-critical operation, such as ciphering or integrity verification; $\mathrm{StateCompat}(A_{i},A_{j})$ indicates that the state machines can be unified without state-variable race conditions; and $\mathrm{StrictDelay}(A_{i},A_{j})$ flags hard real-time latency limitations. The compatibility check additionally preserves PDU/SDU ordering, retransmission behavior, layer-specific inputs and outputs, and failure handling. This rule-based eligibility check vetoes a probabilistic ComBERT candidate whenever any operational constraint fails.

By applying the ComBERT model to process the textual Description of UP PDCP and RLC components, a functional relevance matrix, *M*, between components can be derived:

(10) $$M\left(A_{i},A_{j}\right)=\mathrm{ComBERT}\left({\mathrm{Description}}_{i},{\mathrm{Description}}_{j}\right)$$

Here, $M(A_{i},A_{j})$ denotes the similarity score between components $A_{i}$ and $A_{j}$, computed from their textual descriptions via ComBERT (Algorithm 1). The similarity computation has a complexity of $O(n^{2})$ for *n* components, while each inference scales linearly with text length, making the approach tractable in practice.

**Computational Complexity and Scalability Analysis:** The computational complexity of calculating the pairwise similarity matrix *M* for *n* extracted components is $O(n^{2}\cdot L)$, where *L* denotes the sequence length processed by ComBERT through the sliding-window mechanism. Because component extraction and similarity evaluation occur offline during RAN design and periodic orchestration phases, this step introduces no per-packet latency into the real-time data plane. During runtime, packet forwarding relies on the pre-compiled and optimized service-execution chain. For larger component repositories, embeddings can be cached, and candidate pairs can be pruned by layer, interface, and state compatibility before neural similarity inference.
**Algorithm 1** UP Component similarity algorithm  1:**Input:** Component set $A=\{A_{1},A_{2},\ldots ,A_{n}\}$, ComBERT Model  2:**Output:** Component Similarity Matrix *M*  3:Initialize the similarity matrix *M*, Two-dimensional vector *V*, win_vector  4:**for** $A_{i}\in A$ **do**  5:    text← Extract the text vector representation of component $A_{i}$  6:    Window← Segment the text using a sliding window  7:    **for** w∈Window **do**  8:        Obtain the vector representation of each window using ComBERT w_vector  9:        win_vector.append(w_vector)10:    **end for**11:    $V_{i}\leftarrow$ Aggregate window features max(win_vector)12:**end for**13:**for** i=1 to *n* **do**14:    **for** j=1 to *n* **do**15:        $M\left[i,j\right]=\mathrm{Cosine}\;\mathrm{Similarity}\left(V_{i},V_{j}\right)$16:    **end for**17:**end for**18:* *19:**return** *M*

Based on *M*, Algorithm 2 performs component fusion by grouping components whose similarity exceeds a threshold, θ. Components without qualified matches remain independent, and processed components are marked to ensure determinism. The threshold θ balances fusion granularity and functional independence. Smaller values increase fusion opportunities, whereas larger values preserve modular separation.
**Algorithm 2** UP component fusion algorithm  1:**Input:** Component set $A=\{A_{1},A_{2},\ldots ,A_{n}\}$, Similarity Matrix *M*, Fusion Threshold θ  2:**Output:** Fused Component Set *F*  3:Initialize *F*, Processed Component Set *P*  4:**for** $A_{i}\in A$ **do**  5:    **if** $A_{i}\in P$ **then**  6:        **continue**  7:    **end if**  8:    $S_{i}\leftarrow \left\{A_{j}|M\left[i,j\right]\geq \theta ,\;j\neq i,\;A_{j}\notin P\right\}$  9:    $S_{i}\leftarrow \left\{A_{j}\in S_{i}|\mathrm{ConstraintCheck}\left(A_{i},A_{j}\right)=\mathrm{true}\right\}$10:    **if** $S_{i}\neq \emptyset$ **then**11:        $\hat{c}\leftarrow \mathrm{Merge}\;\mathrm{Component}\left(c_{i},S_{i}\right)$12:        $F\leftarrow F\cup \{\hat{c}\}$13:        $P\leftarrow P\cup \left\{A_{i}\right\}\cup S_{i}$14:    **else**15:        $F\leftarrow F\cup \{A_{i}\}$16:        $P\leftarrow P\cup \{A_{i}\}$17:    **end if**18:**end for**19:* *20:**return** *F*

## 6. Experimental Results and Analysis

The evaluation considers three reference approaches: TF-IDF with cosine similarity as a lexical baseline, standard BERT-base without telecom-domain adaptation, and SBERT as a strong general-purpose sentence-embedding baseline. These methods are compared with ComBERT using regression errors and correlation metrics on the same protocol text-pair test set. At the system level, SBERT-driven and ComBERT-driven fusion are compared under identical simulation assumptions, followed by a sensitivity analysis of the fusion threshold.

### 6.1. Results of Model Pre-Training and Fine-Tuning

**Figure 3:**
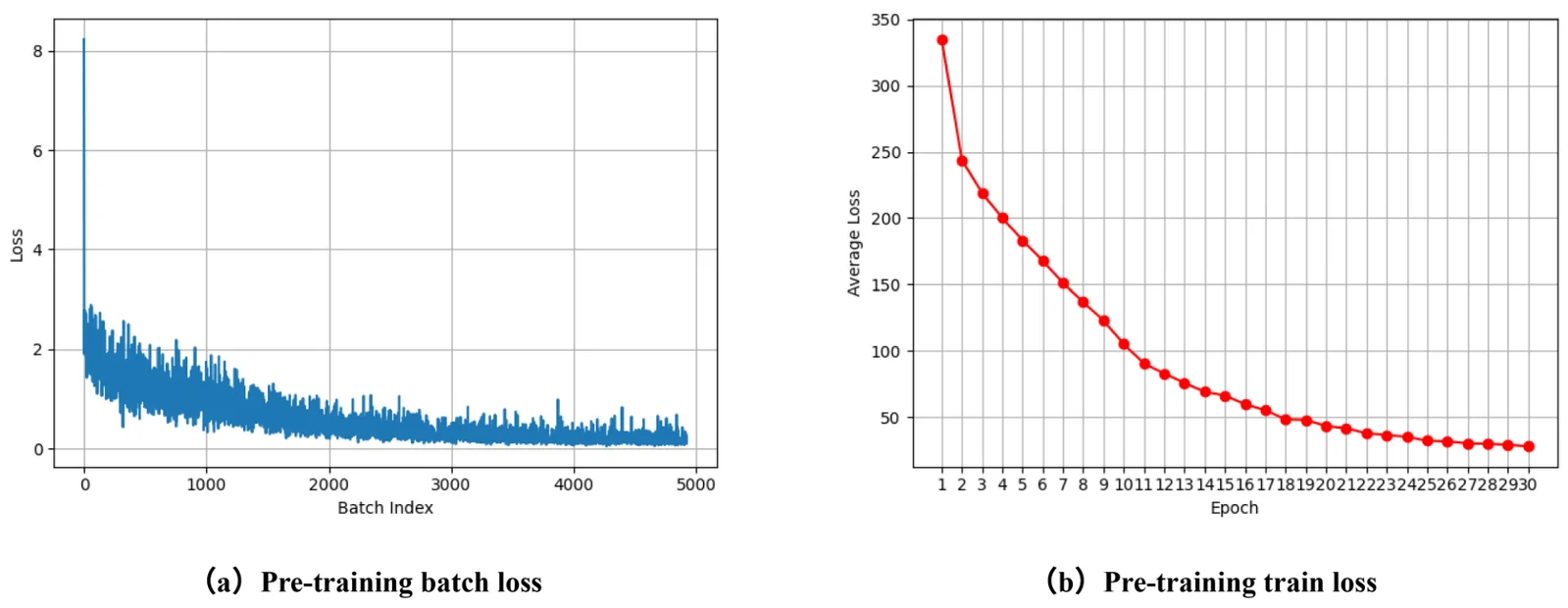
BERT pre-training results.

The parameters used in the pre-training are listed in Table 2. During the pre-training of the BERT model on telecommunication-domain knowledge, this work utilized the 3GPP protocol texts as the training dataset and conducted training based on the MLM task. The training results are presented in Figure 3, where (a) and (b) illustrate the variations in the batch loss and training loss, respectively. From these two subfigures, it can be observed that the loss value decreases gradually as training progresses. This suggests that the model was engaged in continuous learning, progressively refining its parameters to enhance its adaptation to the textual characteristics of the telecommunications domain. Although loss convergence does not fully reflect semantic understanding, it indicates that the model has effectively adapted to the communication-domain text distribution.

All model pre-training and fine-tuning experiments were conducted on a dedicated server equipped with an NVIDIA RTX 4090 GPU with 24 GB of VRAM, an Intel Core i9-13900K CPU, and 64 GB of RAM. ComBERT pre-training used the AdamW optimizer with a linear learning-rate warm-up strategy. The domain-adaptive pre-training phase took approximately 4.2 h over 50 epochs, while downstream text-matching fine-tuning converged within approximately 1.5 h over 70 epochs.

Specifically, after 30 training epochs, the training loss decreased from 340 to below 5, while the batch loss dropped from 8.3 to nearly 0. This trend indicates that the model gradually reduced prediction errors and improved its ability to understand and generate text within the communication domain. The rapid decline in training loss during the early stages of training suggests that the model swiftly learns the fundamental patterns present in the data. As training continues, the model shifts its focus toward capturing more complex features, resulting in a slower rate of loss reduction and ultimately leading to convergence. This behavior is consistent with the typical learning dynamics observed in deep learning models. The trend suggests that the model has achieved a high level of proficiency in the communication domain during pre-training, which enhances its ability to process textual data within this specific domain effectively.

**Figure 4:**
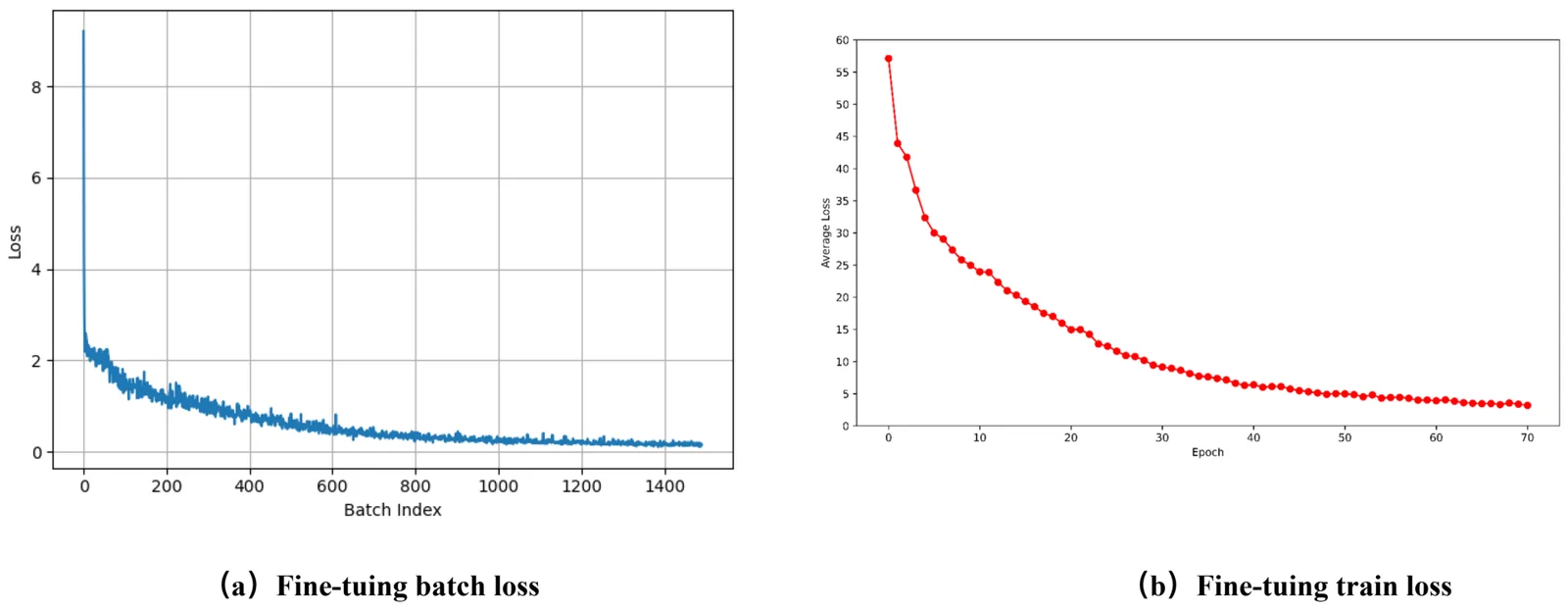
BERT text-matching degree tuning results.

The BERT model was employed for training on the text-matching degree task, with training parameters configured as shown in Table 3. The results are presented in Figure 4. A notable downward trend in loss was observed, indicating that the model was progressively learning and optimizing its predictive capabilities. From the batch loss graph, the loss value rapidly declined from an initial 8.9 and gradually approached zero towards the end of training. This suggests that the model’s performance on individual batches became increasingly stable with diminishing errors. Concurrently, the training loss graph revealed that the average loss decreased significantly from 5.8 to below 0.5 as the number of training epochs increased, further validating the overall improvement in model performance. These results suggest that the ComBERT model is capable of capturing semantic relationships between protocol components and supporting similarity-oriented UP reconstruction tasks. It can effectively minimize the Mean Squared Error (MSE), thereby improving the accuracy of regression tasks.

To evaluate similarity-prediction performance quantitatively, Table 4 reports the Mean Squared Error (MSE), Mean Absolute Error (MAE), Pearson correlation (ρ), and Spearman rank correlation ($r_{s}$). Relative to SBERT, ComBERT reduces MSE by 38.0% and MAE by 39.1% while increasing Pearson and Spearman correlations by 0.041 and 0.052, respectively. Relative to standard BERT, the MSE reduction reaches 63.9%. These results indicate that domain-adaptive pre-training improves both score calibration and pairwise ranking for protocol semantics. Loss convergence in Figure 3 and Figure 4 is used only as an optimization diagnostic; the baseline metrics in Table 4 provide direct evidence of model-level improvement.

**Qualitative Error and Retrieval Analysis:** Representative retrieval outcomes further illustrate the role of domain semantics and operational filtering:

- **True Positive (Correct Fusion Candidate):** PDCP Sequence Number Maintenance and RLC Sequence Number Maintenance obtained $M(A_{i},A_{j})=0.92$. ComBERT recognized that, despite their different sublayer contexts, both components maintain state variables for packet-order tracking.
- **False Positive (Vetoed by Protocol Constraints):** PDCP Duplicate Discarding and RLC Duplicate Detection obtained a high semantic-similarity score of 0.86 because of overlapping terminology. The pair was nevertheless rejected by constraint filtering because the two procedures retain distinct layer-specific state-synchronization and retransmission boundaries.

### 6.2. Results of UP Decoupling and Reconstruction

We integrate discrete-event simulation with probabilistic modeling to emulate the protocol behavior of the PDCP and RLC layers across eMBB, URLLC, and mMTC scenarios within the 5G protocol stack. The objective of the simulation is comparative evaluation under consistent assumptions rather than exact reproduction of commercial RAN deployments. The experiment uses hierarchical modeling to emulate protocol behavior, where component delays are generated based on typical processing characteristics defined in 3GPP TS 38.323 [24] and TS 38.322 [25]. Random events, state transitions, and timestamp recording are incorporated to simulate packet processing. Each experiment is repeated with different random seeds, and the reported results are averaged across runs. The component-delay results are summarized in Table 5. The simulation aims at comparative evaluation under consistent assumptions rather than absolute performance measurement, and focuses on validating the relative effectiveness of semantic-driven UP reconstruction.

Here, $M(A_{i},A_{j})$ denotes the textual matching score between components $A_{i}$ and $A_{j}$, representing their functional relevance. It is computed by feeding ${\mathrm{Description}}_{i}$ and ${\mathrm{Description}}_{j}$ into ComBERT (see Algorithm 1). The similarity computation has complexity $O(n^{2})$ for *n* components, while each inference scales linearly with text length, making the approach tractable in practice.

Based on the relevance matrix, Algorithm 2 fuses PDCP and RLC components. It traverses the component set once, grouping unprocessed components whose similarity exceeds a threshold θ, while retaining isolated ones. All processed components are marked to ensure determinism. The threshold θ controls the trade-off between fusion granularity and reliability.

In the component processing time simulation, protocol functions generate random delays based on standard distributions, with parameters reflecting typical processing characteristics. Network anomalies (e.g., packet loss and protocol errors) are modeled probabilistically, timer thresholds follow normal distributions, and state is maintained via sequence counters and queues. Protocol reconstruction is triggered periodically. PDCP handles direction selection, duplication detection, and encryption, while RLC manages sequencing and segmentation. Reliability is maintained through ARQ, error detection, and status reporting.

The simulation integrates random events, state machines, and timing records to emulate protocol behavior and capture delay distributions. The results provide quantitative insights into key functions such as PDCP encryption and RLC reassembly. The reconstructed UP service units are listed in Table 6. The fusion process only applies to functionally compatible auxiliary procedures, while critical protocol functions with strict timing, security, or state consistency requirements remain independent. The design preserves critical functions (e.g., retransmission and security) while reducing redundancy.

In the fusion process, core PDCP and RLC functions (e.g., encryption, integrity protection, segmentation, and reassembly) remain independent due to technical and security constraints. In contrast, auxiliary processes such as sequence management, duplicate handling, SDU discard, and status reporting are unified through cross-layer optimization. The fused components serve as building blocks for scenario-specific orchestration, where different combinations are selected to construct processing chains.

**Figure 5:**
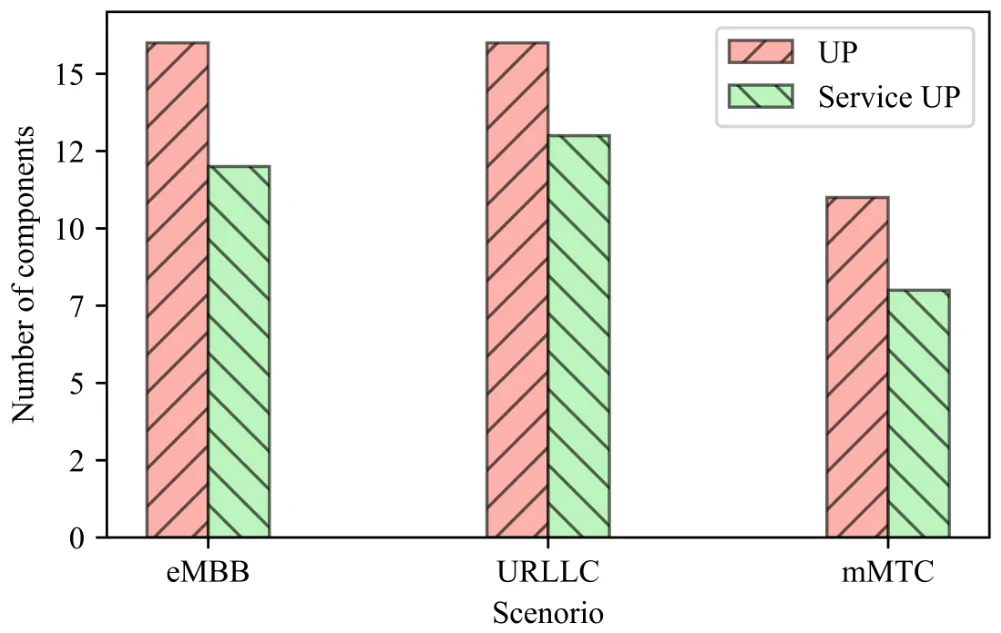
Quantity change diagram of eMBB, URLLC, mMTC components.

Figure 5 illustrates the reduction in component count after UP integration. Compared to the pre-integration configuration, the count decreases by 12.5% in eMBB, 18.75% in URLLC, and 18.2% in mMTC.

**Figure 6:**
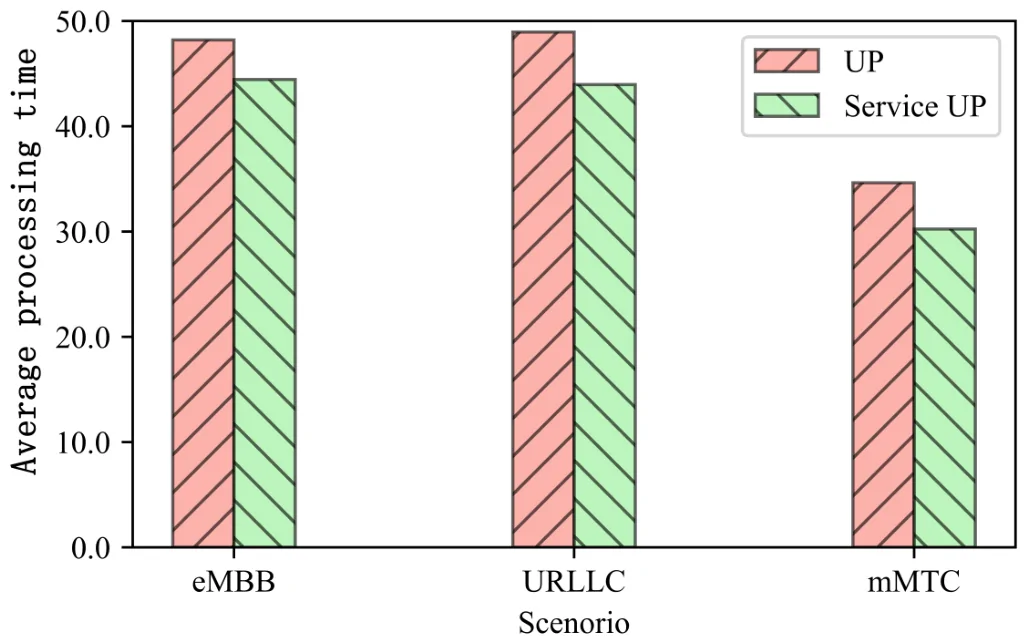
Average processing delay of eMBB, URLLC, mMTC.

As shown in Figure 6, the integrated service-based UP reduces average processing delay by 7.9%, 10.2%, and 11.0% for eMBB, URLLC, and mMTC, respectively. This reduction directly benefits the orchestration process, as fewer fused units need to be composed into processing chains. The reconstructed UP preserves key protocol properties, including retransmission, state consistency, and security, while maintaining scalability through parallelizable similarity computation.

Table 7 compares the system-level component- and delay-reduction results of SBERT-driven and ComBERT-driven fusion.

ComBERT improves the component-reduction rate over SBERT by 3.10, 4.55, and 5.10 percentage points for eMBB, URLLC, and mMTC, respectively. The corresponding delay-reduction improvements are 3.7, 4.4, and 4.9 percentage points. These results indicate that improved protocol-semantic matching consistently translates into lower orchestration complexity and processing delay.

Table 8 shows the expected trade-off. Low thresholds admit more weak candidates and consequently more constraint vetoes, whereas high thresholds miss compatible pairs. The setting θ=0.85 matches the highest reduction observed at θ=0.80 while producing no violations of the modeled constraints and is, therefore, selected as the operating point.

### 6.3. Practical Deployment Limitations and Protocol Generalization

For *n* components, exhaustive similarity construction requires n(n−1)/2 pair evaluations and $O(n^{2})$ matrix storage, while pair inference additionally scales with the number of sliding windows. While tractable for the 22 components evaluated here, practical deployment across broader protocol sets requires embedding caching, exploitation of matrix symmetry, and layer-based pair pruning before neural inference. Runtime packet forwarding does not invoke ComBERT; only the constraint-screened fusion plan and pre-compiled service-execution chain are deployed.

Regarding protocol generalization, while ComBERT is pre-trained on 3GPP specifications (TS 38.323 [24] and TS 38.322 [25]), applying it to non-3GPP specifications (e.g., IEEE 802.11 [26] or IETF protocols) presents specific potential limitations. First, non-3GPP standards often adopt vastly different terminology styles, document structure conventions, and state-machine abstractions, which may cause domain-shift issues for the pre-trained domain tokenizer and embeddings. Second, protocols outside 3GPP may lack highly structured sublayer definitions, making fine-grained component extraction more ambiguous. Extending ComBERT to heterogeneous protocols would thus require multi-domain text corpora for continual pre-training and domain-specific constraint predicates.

Finally, concerning protocol correctness, the proposed Boolean predicate acts as a deterministic safety filter to reject candidates that violate the modeled constraints; it is not a substitute for formal conformance verification. Nevertheless, this work primarily focuses on candidate generation and architectural feasibility. The remaining limitations include the current absence of a physical hardware RAN testbed and formal conformance verification of the fused implementations.

## 7. Conclusions

Motivated by the strict efficiency and agility demands of emerging 6G services, including AI-agent applications, this paper investigates fine-grained UP decoupling for service-based RAN, addressing cross-layer redundancy and limited flexibility in traditional protocol stacks. A domain-specific language model (ComBERT) is developed to capture semantic relationships among PDCP and RLC components. Based on semantic similarity, the method fuses cross-layer redundant components into consolidated service units that serve as building blocks for scenario-specific orchestration, enabling lightweight UP reconstruction.

The model-level comparison shows that ComBERT achieves the lowest MSE and MAE and the highest Pearson and Spearman correlations among TF-IDF, standard BERT, SBERT, and ComBERT. The system-level comparison demonstrates consistent component-count and processing-delay improvements over SBERT-driven fusion, while the threshold analysis identifies θ=0.85 as a balance between fusion opportunities and constraint vetoes. These findings support the feasibility of constraint-aware semantic reconstruction.

**Limitations and Scope of Claims:** AI-agent services provide the architectural motivation for this work, but the experiments do not constitute an application-level evaluation of such services. The eMBB, URLLC, and mMTC traffic profiles are used only to quantify protocol-level component reduction and simulated processing delay. The simulator does not model sensing-data generation, edge-inference latency, control-loop deadlines, or task-level reasoning and performance. Therefore, no conclusion is drawn regarding end-to-end AI-agent service performance. Future work will evaluate the proposed architecture using validated agent-task traces and a physical cloud-native RAN testbed, incorporate formal protocol-conformance checking, and examine generalization to protocols beyond the evaluated 3GPP specifications.

## Figures and Tables

**Table 1:** PDCP and RLC components.

User Plane Protocol	Function		
PDCP	SN Maintenance	Encryption and Decryption	SDU Discard (Timer)
	In-sequence Delivery	Routing	Header Compression
	Integrity Protection	Duplicate Discarding	PDCP Status Reporting
	Duplication	Reordering	PDCP Reestablishment
	Uplink Compression		
RLC	SN Maintenance	SDU Segmentation	SDU Reassembly
	ARQ Error Correction	RLC Reestablishment	SDU Discard
	Protocol Error Detection	Duplicate Detection	Status Reporting

**Table 2:** Pre-training parameter setting.

Parameter	Parameter Value
num train epochs	50
batch size	32
learning rate	5 × 10^−5^
num attention heads	12
num hidden layers	12
hidden size	768
freeze layers	6

**Table 3:** Fine-tuning parameter setting.

Parameter	Parameter Value
num train epochs	70
batch size	256
learning rate	5 × 10^−5^
MLP hidden layers	2
hidden size	4096

**Table 4:** Model-level semantic similarity performance comparison. Downward and upward arrows indicate that lower and higher values are better, respectively. Bold values indicate the best result for each metric.

Model	MSE ↓	MAE ↓	Pearson ρ ↑	Spearman $r_{s}$ ↑
TF-IDF + Cosine	0.246	0.382	0.412	0.395
Standard BERT (Base)	0.158	0.291	0.623	0.608
Sentence-BERT (SBERT)	0.092	0.184	0.846	0.821
**ComBERT (Ours)**	**0.057**	**0.112**	**0.887**	**0.873**

**Table 5:** Delay simulation results of UP components.

PDCP Component	Average Delay (ms)	RLC Component	Average Delay (ms)
SN Maintenance	0.001	SN Maintenance	0.001
Header Compression	0.989	SDU Segmentation	0.651
Uplink Compression	0.993	SDU Reassembly	0.991
Encryption and Decryption	0.581	ARQ Error Correction	5.001
Integrity Protection	0.518	SDU Discard	5.019
Duplication	1.006	Protocol Error Detection	0.037
SDU Discard (Timer)	5.005	Duplicate Detection	1.517
Duplicate Discarding	0.001	Status Reporting	7.139
Reordering	0.903	RLC Reestablishment	20.880
Out-of-Order Delivery	0.986		
PDCP Status Reporting	6.648		
PDCP Reestablishment	20.752		
Routing	0.001		

**Table 6:** Resulting fused and independently retained UP service units.

Fused User Plane Components		
SN Maintenance	PDCP Duplication	SDU Discard
Status Reporting	PDCP Reestablishment/Data Recovery	Encryption and Decryption
Uplink SDU Compression and Decompression	Header Compression and Decompression	Integrity Protection and Verification
Routing	PDCP Duplicate Discarding	RLC Duplicate Detection
Reordering and In-sequence Delivery	RLC Reestablishment	ARQ Error Correction
SDU Segmentation	SDU Reassembly	Protocol Error Detection

**Table 7:** System-level comparison between SBERT- and ComBERT-Driven Fusion. Bold values indicate the best result for each metric within each scenario.

Scenario	Component Reduction		Delay Reduction	
	SBERT	ComBERT	SBERT	ComBERT
eMBB	9.4%	**12.50%**	4.2%	**7.9%**
URLLC	14.20%	**18.75%**	5.8%	**10.2%**
mMTC	13.10%	**18.20%**	6.1%	**11.0%**

**Table 8:** Sensitivity analysis of the fusion threshold in the URLLC configuration. Bold values indicate the selected operating point.

θ	Candidates	Vetoed by Constraints	Component Reduction	Delay Reduction
0.70	9	5	12.50%	6.5%
0.75	8	3	15.62%	8.1%
0.80	7	1	18.75%	10.2%
**0.85**	**6**	**0**	**18.75%**	**10.2%**
0.90	4	0	12.50%	6.4%
0.95	1	0	3.12%	1.8%

## Data Availability

Data will be made available on request.
